# Ovule development, a new model for lateral organ formation

**DOI:** 10.3389/fpls.2014.00117

**Published:** 2014-03-27

**Authors:** Mara Cucinotta, Lucia Colombo, Irma Roig-Villanova

**Affiliations:** Dipartimento di Bioscienze, Università degli Studi di MilanoMilan, Italy

**Keywords:** Arabidopsis, ovule primordia, ovule number, development, transcription factors, hormones

## Abstract

In spermatophytes the ovules upon fertilization give rise to the seeds. It is essential to understand the mechanisms that control ovule number and development as they ultimately determine the final number of seeds and, thereby, the yield in crop plants. In *Arabidopsis thaliana*, ovules arise laterally from a meristematic tissue within the carpel referred to as placenta. For a correct determination of the number of ovules, a precise establishment of the positions where ovule primordia emerge is needed, and a tight definition of the boundaries between ovules is therefore also required. In the last decades, few factors have been identified to be involved in the determination of ovule number. Recently, plant hormones have also been revealed as fundamental players in the control of the initiation of ovule formation. In this review we summarize the current knowledge about both the molecular and hormonal mechanisms that control ovule formation in *Arabidopsis thaliana*.

## Introduction

Fruits are a major evolutionary acquisition of flowering plants (Angiosperms). They likely evolved to protect the developing seeds and to ensure seed dispersal (Knapp, 2002). Fruits derive mostly from the fertilized mature gynoecium although, especially in fleshy fruits, additional floral components have frequently been recruited. The gynoecium (or pistil), the female reproductive organ, is composed of a single carpel or a number of carpels that are often fused. Carpels are essential for sexual plant reproduction because they house the ovules and upon fertilization the carpel develops into the fruit that protects, nourishes and ultimately disperses the seeds.

**Figure 1:**
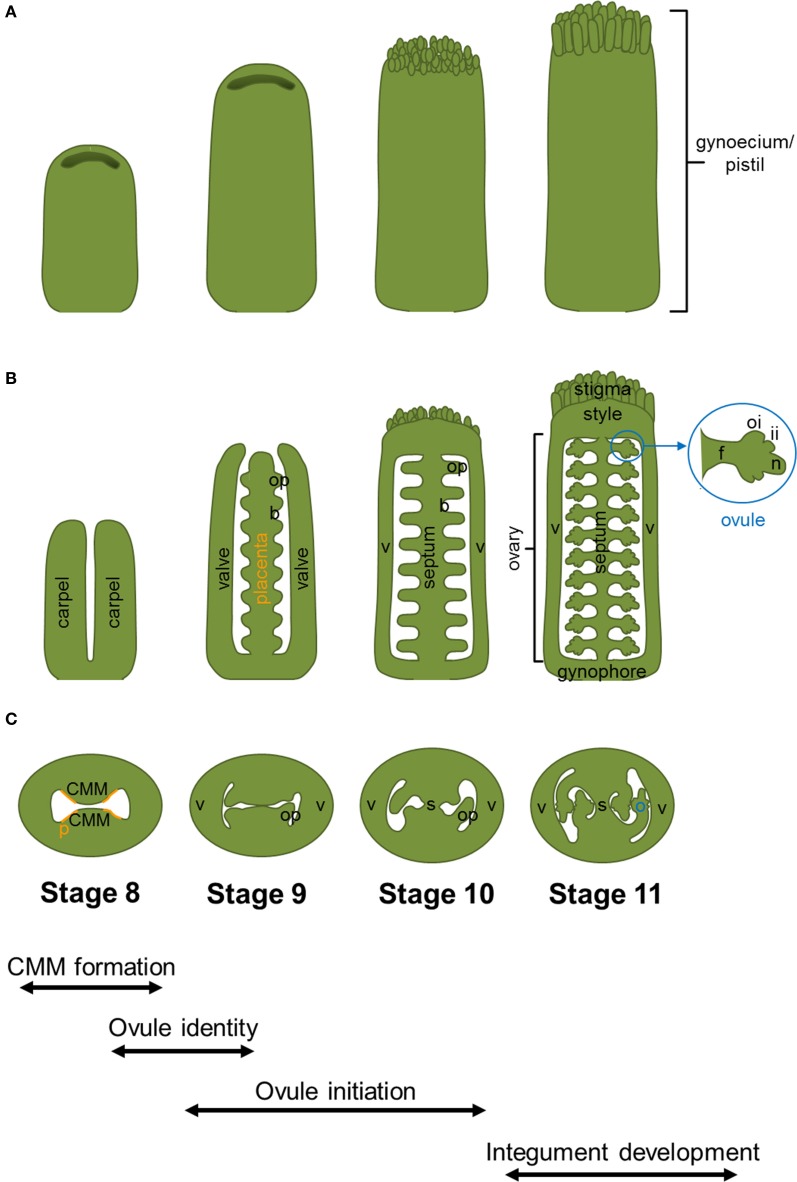
***Arabidopsis thaliana* gynoecium development**. Cartoons displaying wild-type gynoecia **(A)**, in longitudinal sections **(B)**, and transversal sections **(C)** from stage 8 to 11. The different stages and processes of gynoecium and ovule development are indicated at the bottom of the figure. Abbreviations: b, boundary; CMM, carpel margin meristem; f, funiculus; ii, inner integument; n, nucellus; o, ovule; oi, outer integument; op, ovule primordium; p, placenta; s, septum; v, valve. The region of the CMM where placenta is formed is indicated with orange lines.

In Arabidopsis, the fundamental processes leading to the formation of a complete developed set of ovules can be summarized in a few main steps (Figure 1). First of all, the lateral margins of the carpels, containing a meristematic tissue named the medial ridge or carpel margin meristem (CMM), give rise to the placenta, the septum and transmitting track. The CMM formation is known to be controlled by the interaction of genetic and hormonal networks (reviewed by Reyes-Olalde et al., 2013). Once the placenta is formed, some mechanisms, still poorly understood, are needed for the definition of boundary regions that will separate the ovule primordia. The ovule primordia are initiated by periclinal divisions from the subepidermal tissue of the placenta. During the early growth phase of primordia formation a series of predominantly anticlinal divisions take place. Later on, the relatively homogenous mass of cells of the primordium will be organized in three different regions along the proximal-distal axis: the funiculus, the chalaza and the nucellus (Figure 1). Within the nucellus, megasporogenesis and megagametogenesis take place, and finally the mature haploid embryo sac is formed. From the chalaza region the two integuments, progenitors of the seed coat, develop, while the funiculus connects the ovule to the mother plant.

In the last decades, several studies have identified genes involved in ovule identity determination and development in different species such as Arabidopsis, Petunia and rice (Bowman et al., 1991; Angenent et al., 1995; Colombo et al., 1995; Angenent and Colombo, 1996; Pinyopich et al., 2003; Dreni et al., 2007). However, the players that determine the number of ovules are largely unknown, due to the difficulties that the studies tempting to genetically dissect ovule initiation and development have encountered. On one hand, many genes that control ovule development are also involved in initiation and growth of other floral organs, masking their effects on ovules. On the other hand, it is difficult to establish if a mutation in a gene causes a reduction in ovule number in mutants that already display an altered gynoecium phenotype.

Nonetheless, understanding the factors that control ovule initiation is of great importance from an agricultural and economical point of view, as the ovule number will determine the number of seeds that develop in a fruit, and thus the crop yield.

With this review on ovule initiation we aim to summarize the current knowledge about the factors and the hormonal pathways that have been identified to be involved in the determination of ovule numbers in *Arabidopsis thaliana*, and the cross-talk between these hormonal and regulatory pathways.

## Carpel margin meristem formation

### Genetic factors controlling carpel margin meristem formation

As already mentioned, the establishment and maintenance of the meristematic tissues of the CMM is inherently correlated to the generation of ovule primordia. CMM development is known to be controlled at the transcriptional level and by hormones as reviewed by Reyes-Olalde et al. (2013). Several single and higher order mutant combinations with strongly reduced carpel marginal tissue development have been described in literature. One of the them is the *aintegumenta* (*ant*) mutant. ANT is a transcription factor that contains two AP2 domains that controls organ initiation and promotes cellular divisions during organ development (Klucher et al., 1996). Interestingly, the *ant-9* mutant has medial ridges that are frequently unfused to each other with a consequent reduction in functional CMM tissue. It has also been reported that the *ant* mutant displays enhanced morphological defects when combined with a mutation in *REVOLUTA* (*REV*), a member of the class III Homeodomain-Leucine Zipper (HD-ZIP III) family. In the *ant rev* double mutant a partial disruption of CMM and placenta development causes the reduced development of ovule primordia (Nole-Wilson et al., 2010).

An unfused carpels phenotype due to the compromised fusion between the two medial ridges was also observed in the mutants for *LEUNIG* (*LUG*), a floral organ identity gene that encodes a glutamine-rich protein with seven WD repeats, typical of transcriptional co-repressors (Liu et al., 2000). Despite this failure in ridge fusion, ovules are formed from the placenta although in a markedly decreased number in both *lug-1* (intermediate-strength allele) and *lug-3* (strong allele) mutants (Table 1). The simultaneous loss of *LUG* and *ANT* functions enhanced the defects in flower development in respect to the single *lug* and *ant* mutants. While the double mutant *lug-3 ant-9* did not form any ovules, septum or stigma, nearly 50% of the *lug-1 ant-9* pistils could develop normal medial ridges, that gave rise to partially formed septal tissues, although ovules, stigma and style were never present (Liu et al., 2000) (Table 1).

**Table 1:** **Ovule numbers phenotype of the mutants cited in these article**.

**Genotype**	**Ovule number per fruit**	**Ovule number per carpel**	**References**
L*er*		26.4 ± 1.3	Liu et al., 2000
*lug-1*		15.4 ± 4.2	
*lug-3*		14.9 ± 3.1	
*ant-9*		14.8 ± 3.1	
*lug-1 ant-9*		0.0 ± 0	
*lug-3 ant-9*		0.0 ± 0	
Col-0		25 ± 2.0	Azhakanandam et al., 2008
Col-*gl*		21 ± 3.0	
*ant-1*		12 ± 1.3	
*ant-3*		20 ± 2.7	
*seu-3*		23 ± 1.8	
*seu-3* ant-1		0.0 ± 0.0	
*seu-3* ant-3		13 ± 3.4	
Col-0	55.66 ± 0.83		Nahar et al., 2012
*spt-2*	48.38 ± 0.61		
*cuc1-1 spt-2*	36.44 ± 0.59		
*cuc2-1 spt-2*	34.31 ± 0.49		
Col-0	~30		Ishida et al., 2000^*^
*cuc1*	~31		
*cuc2*	~32		
*cuc1cuc2*	~10		
*Ler*	51.8 ± 0.6		Galbiati et al., 2013
*ant-4*	17.8 ± 0.7		
cuc2-1 ant-4^**^	20 ± 3		
*cuc2-1 pSTK::CUC1_RNAi*	41.7 ± 0.9		
cuc2-1 ant-4 pSTK::CUC1_RNAi^**^	8 ± 1		
*pin1-5*	8.6 ± 2		
*Ler*	39.9 ± 1.1		Elliott et al., 1996
*ant-9*	15.0 ± 0.8		
*hll-1*	10% less than *wt*		Skinner et al., 2001
*hll-3*	10% less than *wt*		
L*er*	54 ± 4		Broadhvest et al., 2000
*sin-2*	33 ± 7		
Col-0	48		Bencivenga et al., 2012
*cre1-12 ahk2-2 ahk3-3*	5.5		
*pin1-5*	9.35		
Col-0	110		Bartrina et al., 2011^***^
*ckx3-1 ckx5-1*	65		
Col-0	52.95		Huang et al., 2012
*bzr1-1D*	68.06		
*bin2*	29.07		
*det2*	52		
WS	46.4		
*bri1-5*	32.2		
*ap2-5*	60.4		
*bzr1-1D ap2-5*	74.8		
Cvi	55.5 ± 5.2		Alonso-Blanco et al., 1999
L*er*	66.4 ± 3.9		
*ashh2-1,*	80% less than *wt*		Grini et al., 2009
*ashh2-2,*			
*ashh2-5*			

Mutants presenting defects in the gynoecia or ovule development also reported to be affected at the level of ovule number.

* plants regenerated from calli;

** Galbiati F. personal communication;

*** *the number refers to seeds*.

ANT also interacts synergistically with SEUSS (SEU), a transcriptional coregulator functionally similar to LEU, in the control of organ size of the flower. While the *seu-3* single mutant shows on average ovule numbers not significantly different from wild-type Col-0, the double mutant *seu-3 ant-1* results in a complete loss of ovule initiation, caused by severe defects in early gynoecia development. In the weaker allelic combination *seu-3 ant-3*, employing the *ant-3* hypomorphic allele, placenta formation is not compromised but defects such as ovule initiation and gametogenesis are present at later stages (Table 1) (Azhakanandam et al., 2008).

Other two players in CMM development are CUP-SHAPED COTYLEDON1 (CUC1) and CUC2, two transcription factors that belong to the NAC transcription factor family. The *cuc1* and *cuc2* single mutants display almost no phenotype, while the *cuc1 cuc2* double mutant completely lacks the shoot apical meristem (SAM) and the cotyledons are fused along their margin forming a cup-shaped structure. These seedlings die a few days after germination (Aida et al., 1997). Studying gynoecium development in the *cuc1 cuc2* double mutant was only possible using plants obtained by *in vitro* regeneration. They presented defects in the formation of the septum and in ovule development (Ishida et al., 2000). A gene that has been described to play a role with *CUC1* and *CUC2* in promoting the formation of carpel marginal structures and thus facilitating septum and ovule development is *SPATULA* (*SPT*), which encodes a basic helix–loop–helix (bHLH) transcription factor. Mutations in *SPT* cause a split carpel phenotype in the apical part of the gynoecium. Moreover, *spt* plants have slightly fewer ovules than the wild type, from which only a small fraction develop into seeds (Nahar et al., 2012). When combined with *cuc1* and *cuc2* single mutants, the average number of ovules decreases. Thus, while the *spt* single mutant shows an average of 48 ovules per carpel, *spt cuc1* and *spt cuc2* present 36 and 32 respectively (Table 1), indicating that CUC1, CUC2, and SPT are together required for ovule development. Another mutant that displays an unfused gynoecium at the apex is *crabs claw* (*crc*) (Alvarez and Smyth, 1999). *CRC* encodes a transcription factor of the YABBY family and the characterization of different mutant alleles showed that, besides the failure of the fusion of the stylar region, *crc* mutants present a gradation of phenotypes with wider and shorter gynoecia that contain fewer ovules compared to the wild type (Alvarez and Smyth, 1999; Bowman and Smyth, 1999).

Thereby, the phenotype of ovule reduction that we frequently observe in the mutants defective in medial ridge fusion and thus in CMM formation could be due, at least in part, to their role in regulating cell proliferation in the medial ridges, from which septum and ovules originate.

### CMM formation and the auxin gradient

Auxin is a key hormone for plant development, and it is also fundamental for gynoecium and thereby CMM and ovule development. In the last two decades several studies have demonstrated that local auxin biosynthesis and polar transport are responsible for the correct apical–basal patterning of the gynoecium. The auxin gradient hypothesis supports that high levels of auxin in the gynoecium apical regions control stigma and style formation; medium levels direct ovary formation whereas low levels of the hormone are responsible of gynophore development at the gynoecium base (Nemhauser et al., 2000). Indeed, all mutants in which the auxin synthetic pathway or transport are compromised have a similar severe gynoecium phenotype forming a pistil-like structure with reduction/absence of the valves, expansion of the gynophore and stylar regions and serious vasculature defects (reviewed in Balanzá et al., 2006; Larsson et al., 2013). This phenotype was characterized for the first time in the flowers of *pin-formed1-1* (*pin1-1*), a strong mutant allele of the auxin efflux carrier PIN1 (Okada et al., 1991) and in the *pinoid* mutant, a knock-out line for a serine/threonine kinase that regulates PINs polarity (Bennett et al., 1995). Other examples of mutants with similar pistil-like structure phenotypes are the *yucca1 yucca4* (*yuc1 yuc4*) and *weak ethylene insensitive8 tryptophan aminotransferase related2* (*wei8 tar2*) double mutants, in which local auxin production is impaired (Cheng et al., 2006; Stepanova et al., 2008). Predictably, in most of these auxin-related mutants the severe defects in gynoecium formation lead to a pistil with a reduction or complete absence of ovules and the consequent complete sterility.

Nemhauser et al. (2000) confirmed the importance of polar auxin transport (PAT) in gynoecium development through an experiment in which they used 1-napthylphthalamic acid (NPA), an inhibitor of the auxin transport. They showed that NPA application caused significant loss of ovules. The authors also highlighted that ovules seemed more sensitive to disruption in PAT, with respect to the other tissues of the gynoecium. Indeed, treated carpels were largely devoid of ovules but were still able to produce valves. In 2010 Nole-Wilson and collaborators proposed the connection between ANT and the hormone auxin on the base of the observation that the *ant* mutant is more sensitive than the wild type to alteration in PAT. Moreover, the expression of a subset of auxin-related genes was altered in the *ant* single and *ant rev* double mutant gynoecia, indicating that the morphological defects of the *ant rev* double mutants, at least in part, are due to an alteration in auxin homeostasis in these plants.

Auxin signaling is primarily regulated by the *AUXIN RESPONSE FACTOR* (*ARF*) gene family products, together with the AUXIN/INDOLE-3-ACETIC ACID (AUX/IAA) proteins. The phenotype of *ARF5/MONOPTEROS (MP)* strong mutant alleles results in an embryo lethal phenotype, while *mp* partial loss of function mutants have normal embryo development whereas that their reproductive development is compromised (Hardtke and Berleth, 1998). In the pistil of the *mpS319* weak allele the CMM does not develop, and placenta and ovules are completely missing (Cole et al., 2009; Galbiati et al., 2013). Interestingly, MP has been demonstrated to directly activate the *ANT, CUC1* and *CUC2* transcription factors encoding genes (Galbiati et al., 2013) Their role as major players in ovule primordia initiation and ovule number determination will be discussed in the following sections.

## Ovule identity establishment

The ovule cell fate is controlled by the ovule identity genes *SHATTERPROOF1* (*SHP1*), *SHP2*, and *SEEDSTICK* (*STK*), that belong to the MADS-box gene family of transcription factors. While in the single and double mutant combinations of these genes there is no detectable ovule phenotype, in the *stk shp1 shp2* triple mutant the ovule integuments are converted into leaf/carpel-like structures (Pinyopich et al., 2003; Brambilla et al., 2007). Moreover, ectopic expression of these MADS box genes results in ovule formation on sepals (Favaro et al., 2003; Pinyopich et al., 2003; Battaglia et al., 2006). *STK, SHP1*, and *SHP2* have overlapping expression patterns in the placenta and ovule primordia also with *AGAMOUS (AG)* (Rounsley et al., 1995; Savidge et al., 1995; Theißen et al., 1996; Pinyopich et al., 2003), one of the first identified MADS-box factors that determines stamen and carpel identity (Yanofsky et al., 1990). It has been shown that also AG plays a role in ovule development by experiments in which the *apetala2* (*ap2*) single mutant was compared with the *ap2 ag* double mutant. Thus, in the *ap2* single mutant petals were mostly absent, while sepals were converted into carpel structures bearing ectopic ovules, some of which were transformed into carpelloid structures. Interestingly, the sepals (or first-whorl organs) of the *ap2 ag* double mutant still presented carpel identity, and the number of ovules converted into carpel structures was significantly higher, indicating that AG activity also contributes to ovule identity establishment (Bowman et al., 1991; Pinyopich et al., 2003). Interestingly, Skinner et al. (2004) suggested that when the functions of *stk, shp1*, and *shp2* were lost in a triple mutant, fewer ovules initiated and ovule development is severely disrupted.

## Ovule primordia initiation

### The establishment of the boundaries

When new organ primordia are originated in the plant, two different regions, the boundaries and the zone of primordia outgrowth, need to be defined. The organ boundary is defined as the region between the meristem and the developing organ, or, as in the case of ovules, as the region between two adjacent ovule primordia. As Aida and Tasaka nicely reviewed in 2006, the “boundary cells” need to have peculiar characteristics respect to the surrounding cells, usually displaying reduced cell division and expansion. Another important aspect is the arrangement of the plasmodesmata that regulates the movement of transcription factors between cells. For example, the boundaries in the inflorescence meristem seem to restrict the passage of proteins into flower primordia (Wu, 2003).

The boundary-specific regulatory genes play a critical role in orchestrating several morphogenetic and patterning events and their spatial coordination. When this coordination is missing, fusion between organs is the most frequent observed phenotype (Aida et al., 1997). The *CUC* gene family was the first discovered to have a fundamental role in organ boundary establishment. In fact, in the *cuc1cuc2* double mutant embryo the cotyledons do not separate (Aida et al., 1997).

**Figure 2:**
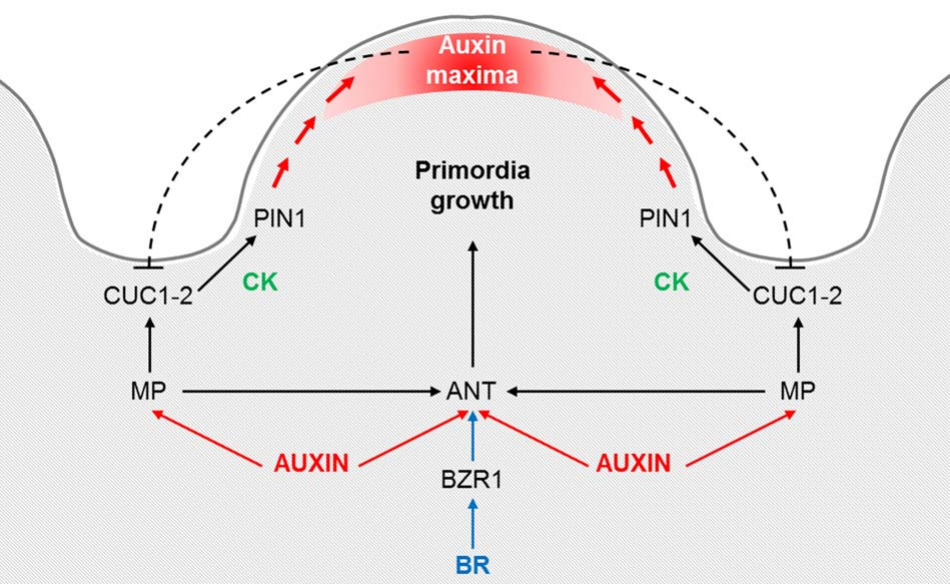
**Proposed model for the control of the ovule primordia initiation**. Auxin triggers ANT and MP expression, which in its turn is required for *ANT, CUC1* and *CUC2* expression during the early stages of placenta development and ovule primordia formation. *ANT* expression is also regulated by brassinosteroids (BR), specifically being directly regulated by BZR1. ANT controls cell proliferation in the placenta and ovules, whereas CUC1 and CUC2 establish the boundaries and control *PIN1* expression, which is required for primordia formation. Cytokinin (CK) may act downstream of CUC proteins in promoting *PIN1* expression. Once the primordia have formed, auxin accumulates at the edge of the developing ovule. An inhibitory loop of auxins on CUC1 and CUC2, as it is postulated for the leaf serration, could be happening at the ovule boundaries. Adapted from Galbiati et al. (2013).

The transcripts of *CUC1* and *CUC2* were detected by *in situ* hybridization in the anlagen placenta and in ovules at stage 1-II and later on, starting from stage 2-I, restricted to the boundary between two ovules (Ishida et al., 2000; Galbiati et al., 2013). As we already mentioned, the study of the gynoecium phenotype of the *cuc1 cuc2* double mutant was only possible on plants regenerated *in vitro*. They showed defects in the formation of the septum and in ovule development; most of the gynoecia having less than 10 ovules (Table 1). However, the *cuc1 cuc2* double mutant plants never gave seeds (Ishida et al., 2000). A further demonstration that CUC1 and CUC2 are directly linked to the determination of ovule number in a direct way came from the work of Galbiati et al. (2013). In order to study the ovule phenotype in absence of both *CUC1* and *CUC2, CUC1* was silenced in a *cuc2-1* mutant background using a *CUC1* specific RNAi construct under the control of the ovule-specific *SEEDSTICK* promoter (*pSTK:CUC1_RNAi*) which is already active in the placenta before ovule primordia arise. The analysis of *cuc2-1 pSTK:CUC1_RNAi* plants revealed a reduction in ovule number of 20% (Table 1). Furthermore, *ant-4 cuc2-1 pSTK::CUC1_RNAi* plants were generated in order to analyze the possible additive role of ANT to CUC function in the regulation of ovule primordia formation. The *ant-4 cuc2-1 pSTK::CUC1_RNAi* plants displayed a further dramatic reduction in the number of developing ovules (a mean of seven ovule primordia per pistil), while the single mutant *ant*-*4* and the plants *cuc2-1 pSTK::CUC1_RNAi* showed 20 and 30 ovules per pistil, respectively (Table 1). Despite the reduction in ovule number in the different mutant backgrounds, the size of the pistils was not reduced. Therefore, the ovules were more distantly spaced compared to those in wild-type pistils (Galbiati et al., 2013). These studies of the characterization of the *ant* single and *cuc1 cuc2* double mutants, as well as *ant-4 cuc2-1 pSTK::CUC1_RNAi* plants prove that ANT, CUC1 and CUC2 are key players in the control of the number of ovule primordia that develop from the placenta and that they act additively (Elliott et al., 1996; Ishida et al., 2000; Galbiati et al., 2013). All the information about these factors taken together indicates that they work in different ways: while ANT promotes ovule primordia growth, the CUCs play a role in the establishment of the ovule primordia boundaries (Figure 2).

*CUC3*, another putative NAC-domain transcription factor member of the *CUC* family, is expressed in an extensive range of boundaries in adult plants. Besides, the function of CUC3 is partially redundant with that of its homologous CUC1 and CUC2 in the establishment of the cotyledon boundary (Vroemen et al., 2003). Several studies revealed that *CUC* expression is controlled and restricted to the boundaries in several ways. For instance, in the SAM *CUC1* and *CUC2* but not *CUC3* are regulated by *miR164*, which restricts the expression of *CUC1* and *CUC2* mRNAs to the boundary domain (Laufs et al., 2004). In the carpel, in a similar way to the pattern already described for *CUC1* and *CUC2, CUC3* expression marks the boundaries between ovule primordia. Therefore, it would be interesting to study also the contribution of *CUC3* in the regulation of defining ovule boundaries.

In 2008, Xu and Shen showed that three different transcription factors, ASSYMETRIC LEAVES 1 (AS1), AS2, and JAGGED (JAG), support normal sepal and petal growth by restricting the expression domain of the boundary-specifying genes *CUC1* and *CUC2*. AS1 and AS2 were already suggested to have roles in boundary control, given that they positive regulate, within the shoot apex, the members of the *LATERAL ORGAN BOUNDARIES* (*LOB*) gene family, a plant-specific family of transcription factors that are expressed in the boundaries (Byrne et al., 2002). *LOB*, the gene that names the family, is expressed at the base of all lateral organs. Interestingly, plants overexpressing *LOB* produced abnormal flowers with reduced floral organs and they were sterile even when fertilized with wild-type pollen (Shuai et al., 2002). Lee et al. in 2009 identified two new MYB transcription factors involved in lateral organ separation: *LATERAL ORGAN FUSION 1* (*LOF1*) and *LOF2*. The single mutant *lof1* exhibits a novel fusion between the axillary stem and the cauline leaf. Additional fusions resulted when *lof1* was combined with *lof2, cuc2* or *cuc3*, indicating the existence of overlapping roles for *LOF1, CUC2*, and *CUC3* to control organ separation during reproductive development.

Despite the identification of a number of boundary-specific transcription factors, boundary formation and maintenance is still a poorly understood process, and only CUC1 and CUC2 have been demonstrated to have a role in ovule boundary establishment. The factors that have been described to regulate or interact with the CUCs in a different developmental context could also have a role during ovule initiation, and some of them, like *AS1* and *AS2* are already known to be expressed in the gynoecium and ovules (Xu and Shen, 2008).

### Aintegumenta, a master regulator of primordia formation

In Arabidopsis many genes have been described to play roles in the different phases of ovule development, although most of them do not determine directly the number of ovules (Schneitz et al., 1997; reviewed in Shi and Yang, 2011). However, the ANT transcription factor has been described to have a clear role in ovule primordia formation. *In situ* hybridization experiments showed that within the carpel it is expressed in the placenta and in the integuments of the developing ovules. In *ant* plants ovules do not develop integuments and megasporogenesis is blocked at the tetrad stage leading to complete female-sterility (Elliott et al., 1996). ANT is not only required for ovule development but it is also involved in ovule primordia formation. Indeed, in the *ant-9* mutant the number of ovules per carpel is reduced by more than half in respect to the wild-type (Table 1). Given that the *ant* gynoecia have the same length as those of wild type, the ovules that do arise in *ant* are more distantly spaced than in wild-type plants (Liu et al., 2000).

In addition to *ANT*, another essential gene for the regulation of ovule primordia outgrowth and for the control of integument formation is *HUELLENLOS* (*HLL*), a gene that encodes a mitochondrial ribosomal protein. Thus, plants presenting mutations in *HLL* display a phenotype similar to *ant* at the level of ovule integuments (Schneitz et al., 1997, 1998). Moreover, *hll-1* and *hll-3* mutant alleles display a reduction of about 10% in the number of ovules, although the authors also describe that *hll* plants display smaller gynoecia, which could contribute to the development of fewer ovules (Table 1). The phenotype of the double mutant *hll ant* was more severe at the level of primordia outgrowth however, nothing was described regarding ovule number (Schneitz et al., 1998; Skinner et al., 2001). A similar phenotype to *hll* was observed in the *short integuments 2* (*sin2*) mutants. Apart of an arrest in cell division in both ovule integuments, *sin2* plants presented shorter pistils bearing less ovules than the wild type (Table 1). Moreover, the authors describe an abnormal distribution of the ovules along the placenta, being the distance between ovules bigger than in wild-type plants (Broadhvest et al., 2000). Thus, in this particular case the shorter carpel might not be the only cause of reduced ovule numbers. The double mutant *sin2 ant-5* was not different from *ant-5* single mutant, indicating that *ANT* is epistatic to *SIN2* with respect to ovule development. On the contrary, *sin2 hll-1* double mutant had a stronger effect on ovule development than *sin2* or *hll-1* single mutants (Broadhvest et al., 2000). All these experiments taken together indicate that although ANT plays a master role, SIN2 and HLL also contribute to ovule primordia formation.

## The role of hormones in ovule primordia formation

### Auxin is required for ovule primordia formation

As we previously underlined, the boundary region and the primordia formation zone are highly interconnected. It has been demonstrated that a fundamental role of the “boundary transcription factors” is to organize PAT, mediated by PIN proteins, in order to create a zone of auxin maximum where organ founder cells will be selected. Auxin maxima are fundamental for the formation of primordia, and auxin action has been well described for lateral roots (LR) and flower primordia (reviewed in Benková et al., 2003, 2009; Yamaguchi et al., 2013). The directionality of auxin flux depends principally on the polar localization of the PIN proteins. In Arabidopsis there are eight PIN proteins (PIN1-8), from which only *PIN1* and *PIN3* are expressed in the pistil and ovules (Benková et al., 2003; Ceccato et al., 2013). PIN1 protein is localized at the membrane of placenta cells and later on, in the developing ovules, it is restricted to the lateral-apical membranes of nucellus cells. PIN3 is also present in few cells at the tip of the developing nucellus shortly after ovule primordia emergence but, contrary to PIN1, it is not expressed in the placenta cells (Ceccato et al., 2013). PIN-dependent efflux mediates primordium development by supplying auxin to the tip creating an auxin maxima; indeed in plants expressing the *GFP* reporter gene downstream the auxin-responsive *DR5* promoter (*pDR5::GFP*), the GFP signal is detected at the tip of all ovule primordia (Benková et al., 2003). The weak *pin1-5* mutant allele is able to develop some flowers in which the pistils have slightly reduced valves but normal styles and stigmas (Sohlberg et al., 2006). The pistils of the *pin1-5* weak allele have an average of 9 ovules per carpel (Table 1) (Bencivenga et al., 2012). In addition, Galbiati et al. (2013) demonstrated that the reduced number of ovules in *cuc2-1 pSTK:CUC1_RNAi* was caused by a down-regulation of *PIN1* and an incorrect PIN1 protein localization. CUC1 and CUC2 promote *PIN1* expression and localization to correctly form the auxin maximum where primordium will form (Figure 2). In the same way, a CUC2-dependent regulatory pathway controlling PIN1-mediated auxin efflux has been described to explain leaf serrations (Bilsborough et al., 2011). Moreover, in the newly formed primordia of the SAM the auxin maxima, in a negative feed-back loop, repress *CUC2* expression and restricts it to the boundaries (Vernoux et al., 2000; Heisler et al., 2005; and reviewed in Aida and Tasaka, 2006; Rast and Simon, 2008). A similar inhibitory loop could control *CUC* expression at the ovule boundaries (Figure 2). The phenotype of *cuc2-1 pSTK:CUC1_RNAi* was completely recovered by cytokinin (CK) application, since CK has been demonstrated to increase *PIN1* expression in the ovules (Bencivenga et al., 2012). These experiments evidence a convergence of two different plant hormones in the regulation of ovule primordia formation. In the next paragraph we will delve deeper into the role of CK in the formation and determination of ovule number.

### Cytokinin positively regulates ovule number

CK is an essential hormone for plant growth and development as it has a central role in the regulation of cell division and differentiation. In the last 10 years, several studies have clearly proven that CK has also a significant role during ovule development. As it will be explained in this paragraph, it has been demonstrated that in plants that are defective in the production or perception of this hormone, correct ovule formation is compromised and/or the number of ovule is drastically reduced. CK signaling, which has been recently summarized in a detailed review article (Hwang et al., 2012), is mediated by a two-component signaling pathway: histidine protein kinases (AHKs) work as CK receptors, while histidine phosphotransfer proteins (AHPs) transmit the signal from AHKs to nuclear response regulators (ARRs), which are able to regulate transcription. In *Arabidopsis* the CK signal is perceived by three histidine kinases: *ARABIDOPSIS HISTIDINE KINASE4* (*AHK4*, also known as *CYTOKININ RESPONSE1, CRE1*/*WOODEN LEG, WOL*), *AHK2* and *AHK3*. These three genes are all expressed in inflorescences, carpels and developing ovules (Higuchi et al., 2004; Nishimura et al., 2004). More precisely, *AHK2* and *AHK3* are expressed during all stages of ovule development, starting from early primordia stages to ovule maturity, whereas *CRE1* expression remains restricted to the chalazal region and later to the integuments of ovules during all the developmental stages (Bencivenga et al., 2012). The single and double mutants of *AHKs* do not present any phenotype at the level of the ovules (Higuchi et al., 2004). However, mutants lacking all three receptors exhibit no perception of CK and present a strong slowdown of shoot and root growth. The resulting miniature plants also show delayed flower induction and impaired fertility (Higuchi et al., 2004; Nishimura et al., 2004; Riefler et al., 2006). Thus, the triple mutant *cre1-12 ahk2-2 ahk3-3* do not produce seeds (Higuchi et al., 2004) because the gametophyte arrests at stage FG1-FG2 (Bencivenga et al., 2012). Moreover, a severe reduction in the ovule number, an average of 5 ovule per pistil, was noticed in these triple mutant plants (Table 1) (Bencivenga et al., 2012). A similar sterile phenotype was also observed for another allelic combination: the *ahk4-1 ahk2-1 ahk3-1* triple mutant (Nishimura et al., 2004). Differently, Riefler et al. (2006) obtained a weaker triple mutant *cre1-2 ahk2-5 ahk3-7* that self-fertilized and formed few seeds, suggesting that infertility of the histidine kinase triple mutants is a phenotype associated with specific mutant alleles.

Attention has also been given to the importance of CK catabolism. In *Arabidopsis* the irreversible degradation of CK is catalyzed by the oxidase/dehydrogenase (CKX). The *CKX* gene family of Arabidopsis consists of seven members (*CKX1* to *CKX7*), and by *promoter:GUS* fusion constructs it was shown that *CKX1, CKX5*, and *CKX6* (At3g63440, previously called *AtCKX7*) are expressed in flower tissues, being *CKX6* the only one reported to be expressed in the carpel and ovules, in particular in the funiculus (Werner et al., 2003). Werner and colleagues engineered transgenic *Arabidopsis* plants that individually overexpressed six different *CKXs* in order to enhance CK degradation. As expected, these plants manifested phenotypes linked to CK deficiency, like delayed vegetative growth and leaf expansion, diminished activity and size of the SAM but increased overall root system. The reproductive development of CK-deficient plants was also altered. In *35S::CKX1* and *35S::CKX3* plants, flowering was strongly delayed and furthermore the fertility of flowers was heavily reduced, partially due to the lack of pollen. *35S::CKX1* and *35S::CKX3* siliques were not filled completely and they formed approximately 8–20 viable seeds, whereas the wild-type siliques harbored up to 60 seeds. Although the number of ovules formed in these plants was not reported in this work, the expression patterns together with the phenotypes in the flowers and fruits indicate once more that CK play a role during reproduction. Moreover, the authors suggest a role for ANT in the observed reduced cell division in the leaves of *ckx* plants. Considering the documented role of ANT in ovule primordia initiation already introduced in this article, it will be very interesting to analyze also its role in the reproductive tissues of these plants.

With an opposite experimental approach, the simultaneous mutations of two *CKX* genes, it was demonstrated that plants with an increased level of CK had an enhanced activity of the reproductive meristem (Bartrina et al., 2011). Indeed, the *ckx3-1 ckx5-1* double mutant produced more flowers due to a larger inflorescence meristem with more cells than the wild type. Moreover, flowers were bigger and so were the gynoecia. Besides, double mutant gynoecia contained twice as many ovules as wild-type ones, indicating an increased activity of their placental tissue. The *ckx3-1* and *ckx5-1* single mutants already developed more ovules than the wild-type, and the flower size and the number of ovules was reflected into the length of the fruits (siliques of *ckx3 ckx5* were 20 mm long compared with the 17 mm of the wild type) and the seed number (110 seeds in the *ckx3 ckx5* mutant siliques compared with an average of 65 seeds in wild-type siliques, Table 1) The authors suggested that CKX3 and CKX5 may regulate the activity of meristematic cells in the placenta thus affecting organogenic capacity and ovule primordia formation.

A conclusive evidence about the relationship between the levels of CK and the initiation of ovule formation was obtained from experiments in which inflorescences were treated with synthetic CK (6-Benzylaminopurine, BAP). The treatment resulted in the formation of new primordia, 20 ± 3 primordia in average in each pistil, positioned between the ovules already formed before the CK application (Bencivenga et al., 2012). An equivalent CK treatment was also able to increase the ovule number in *pSTK::CUC1_RNAi cuc2* plants already described in this review, by acting on the expression and localization of the auxin efflux carrier PIN1 (Galbiati et al., 2013). These results point out the importance of the cross-talk between CK and auxin during ovule primordia formation. However, the hormonal cross-talk is not limited to auxin and CK since very recently it has been demonstrated that also brassinosteroids (BR) play a crucial role in ovule and seed formation by regulating the expression of genes that control ovule development (Huang et al., 2012), as will be explained in the next paragraph.

### The role of brassinosteroids

BRs are hormones known to control general plant development. More specifically, they have been described as involved in the control of the initiation and formation of reproductive organs (Szekeres et al., 1996; Kim et al., 2005). Huang et al. (2012) found that the BR-deficient and -insensitive mutants have smaller and less seeds, while BR-enhanced mutants have more seeds. The analysis of the number of ovules and seeds and the morphological analysis of the siliques of *det-2* (a BR-deficient mutant involved in BR biosynthesis), *bri1-5* (the mutant for the BR receptor), heterozygous plants for *bin2-1* (a gain of function mutant deficient in BR signaling) and *bzr1-1D* (a BR signal-enhanced mutant) leaded to the conclusion that BR signaling positively regulates ovule number (Table 1) (Huang et al., 2012). Specifically, it was found that the transcription factor BRZ1 plays an important role in ovule and seed number determination, depending on its state of phosphorylation/dephosphorylation (more dephosphorylation implying more activity and more ovules and seeds).

By treating plants with BR it was shown that BR influences ovule development through regulating the transcription of genes such as *HLL* and *ANT*, which are redundant in the control of ovule primordia growth as already introduced in this review (Schneitz et al., 1998), and *AP2*, that affects floral organ (including ovule) pattern formation (Modrusan et al., 1994). *HLL* and *ANT* are clearly induced by BR, while *AP2* is slightly repressed by BR. These genes appeared to be targets of BRZ1, and its state of phosphorylation/dephosphorylation influences the expression of these genes. Further analysis indicated that *AP2* and *ANT* are direct targets of BRZ1, while *HLL* is regulated by an indirect way. The analysis of ovule number of *bzr1-1D* and *ap2-5* single mutants and *bzr1-1D ap2-5* double mutant (Table 1), together with other molecular proofs, indicate that BZR1 and AP2 play antagonistic effects in ovule number determination, being BZR1 (and HLL and ANT) promoters and AP2 inhibitor of ovule primordia formation (Huang et al., 2012).

A model for ovule primordia formation that integrates the molecular and hormonal networks has been proposed by Galbiati et al. (2013): MP is required for *ANT, CUC1* and *CUC2* expression during the early stages of placenta development and ovule primordia formation, being ANT expressed in the ovule primordia, whereas *CUC1* and *CUC2* in the ovule boundaries. CUC1 and CUC2 may be involved in the increase of CKs required for proper *PIN1* expression needed for primordia formation. Once the primordia have formed, auxin accumulates at the edge of the developing ovule. This model can be easily extended with the recently discovered role of the plant hormones BR, which positively regulate the number of ovule primordia, in part by the direct regulation of *ANT* by BZR1 (Figure 2).

## Other mechanisms controlling ovule number: the epigenetic regulation

Interestingly, in different Arabidopsis ecotypes (diploid accessions) a variation in ovule numbers can be observed. Alonso-Blanco et al. (1999) found that the Lansberg *erecta* ecotype presents 20% more ovules than the Cape Verde Islands (Cvi) one (Table 1). Recently a considerable genetic variation in ovule number was described in selfed F1 triploids of different *A. thaliana* genotypes (Duszynska et al., 2013). Triploids were obtained by crossing a tetraploid L*er*-0 line (used as a male or female parent) with different diploid accessions. Interestingly, it was observed an effect of the parental genome excess (2m:1p *vs*. 1m:2p) in the determination of the total ovule number in genetically identical F1 hybrid offsprings. These were the first parent-of-origin effects on ovule number in reciprocal triploids of plants. The authors postulate that such effects may represent epigenetic effects, because changes in DNA sequence cannot explain mitotically and/or meiotically heritable changes in gene function but they might be due to changes in DNA methylation, for example (Duszynska et al., 2013). Indeed, in Arabidopsis the ASH1 class of proteins, that can methylate lysine residues on histone tails, maintains an active transcriptional state during development. One of its members, ASH1 HOMOLOG 2 (ASHH2), has been described as a controller of reproductive development via H3K36 trimethylation. Plants homozygous for *ashh2* null alleles presented an 80% reduction in ovule numbers when compared to wild-type plants (Table 1) (Grini et al., 2009). These data altogether indicate that epigenetics may also play a role in the control of ovule number, and they open up a new interesting field of research.

## Concluding remarks and further perspectives

In the past years, several genes such as *AG, STK, SHP1*, and *SHP2* have been identified as ovule identity genes, and *ANT, HLL, SIN2, INNER NO OUTER* (*INO*), and *SUPERMAN* (*SUP*) as regulators of ovule outgrowth (Elliott et al., 1996; Schneitz et al., 1997, 1998; Broadhvest et al., 2000; Pinyopich et al., 2003). Nevertheless, most of their targets, which might be the genes that determine the correct development of the ovule, remain to be uncovered. Another quite unknown process is the regulation of the ovule primordia initiation. As explained in this review, only a few regulators, such as the transcription factors ANT, CUC1, CUC2, AP2 and the mitochondrial ribosomal protein HLL have been identified (Elliott et al., 1996; Schneitz et al., 1998; Galbiati et al., 2013). The majority of them are transcription factors, and the transcriptional cascades triggered by them, that will determine the regulation of the morphogenetic parameters such as cell division and expansion, or expression patterns of identity genes of particular organs, are also largely unknown. Therefore, one of the next challenges would be the identification of downstream targets of these transcription factors by genetic or molecular biological approaches, including suppressor/enhancer mutant screenings or RNA-sequencing transcriptome analyses. It is worth to highlight that these regulators are not exclusively transcription factors, but also mitochondrial proteins or chromatin remodeling factors, indicating that a correct ovule initiation depends on a complex genetic and molecular network.

One of the difficulties of the genetic dissection of ovule initiation and development is that many mutations that affect ovule initiation have already pleiotropic effects on earlier stages of the development of the reproductive tissues, causing floral aberrations that may mask their effects on ovules. Thus, many genes that control ovule development are also involved in primordium initiation and growth of other floral organs (Elliott et al., 1996; Schneitz et al., 1998; Alvarez and Smyth, 1999). Moreover, it is difficult to establish if a mutation in a gene causes a reduction in ovule number if this mutant already has an altered gynoecium phenotype (Alvarez and Smyth, 1999; Western and Haughn, 1999; Broadhvest et al., 2000; Liu et al., 2000; Pinyopich et al., 2003; Nole-Wilson et al., 2010; Nahar et al., 2012). The reanalysis of these carpel mutants, measuring the space between ovules, or expressing the ovule number as the ovule number per millimeter of gynoecium, as some authors already presented (Huang et al., 2012), could contribute to resolve this uncertainty. The use of specialized vectors, for instance containing placenta-specific promoters to obtain milder vegetative and/or floral effects of these mutations would help to uncover the role of specific factors in ovule development. Besides, a reverse-genetic strategy using RNA interference or insertional mutants can be used to identify new regulators of ovule numbers determination.

Ovule boundary establishment is still a poorly understood process, and only CUC1 and CUC2 have been demonstrated to play a role (Galbiati et al., 2013). The contribution to the determination of ovule boundaries of the genes that have been described to regulate or interact with the *CUCs* in other organ boundaries would be worth to be analyzed, by means of the study of their patterns of expression and how these are accurately determined. The identification and characterization of single and multiple mutants, as has been done for the *CUC* genes (Aida et al., 1997; Galbiati et al., 2013) is also key to study their roles. Moreover, the analysis of their incidence at the cellular level will help to define the effects on cell behavior (i.e., division or expansion) that these factors could have. It has also been widely demonstrated that hormones play a role in the regulation of ovule primordia initiation, being auxin, CK and more recently also BR identified as the important hormonal players in this process. The crosstalk between these hormones, as Bencivenga et al. (2012) and Galbiati et al. (2013) present in their works, is starting to be revealed (Figure 2) and it will be very interesting to investigate in the future how auxin, CK and BR interact. Moreover, it will be important to explore if hormone and gene expression levels are responsible for the variation in ovule numbers described for the different ecotypes (Alonso-Blanco et al., 1999), and to identify QTLs linked to this trait.

Based on the experimental data exposed in this review, a similarity between ovule initiation and the initiation of other lateral organs in the plant can be proposed. The strongest pieces of evidence are the triggering role of auxins and the conservation in the genes that establish the boundaries and promote new organ growth. Although further studies will be needed in order to identify the common and specific players of the different lateral organ initiation processes, conserved modules can be already suggested. In the case of flower primordia initiation, similarly to what happens during ovule primordia formation, the coordinate action of MP and ANT is required. In particular, at the reproductive shoot apex, auxin-activated MP directly induces *ANT*, other two key regulators of floral growth, *LEAFY* (LFY) and *AINTEGUMENTA LIKE-6* (*AIL6*), and probably a forth unknown factor, which together lead the flower primordium initiation (Yamaguchi et al., 2013). Also the factors determining the new organ boundary seem conserved between ovule and flower primordia initiation: the coordinated spatial and temporal action of auxin, PIN transporters and CUC proteins is required (Heisler et al., 2005; Galbiati et al., 2013). If we instead compare the initiation of ovule primordia with the initiation of LR we also find many common players, despite the clear fact that ovule primordia arise from the naked placenta while LR have to pass through several cell layers to emerge. Thus, we find an auxin maxima that precedes organ formation (Benková et al., 2003). Also other hormones, such as BR and CK play a role in both ovule and LR initiation, although CK play opposite roles (it activates ovule primordia formation while inhibits LR initiation) (Werner et al., 2003; Higuchi et al., 2004; Bartrina et al., 2011; Huang et al., 2012; Bianco et al., 2013; Chang et al., 2013). Besides, the participation of IAA/AUX-ARF modules exists in both processes, and MP seems to be a regulator of the two of them (De Smet et al., 2010; Galbiati et al., 2013). Other members of the ARF family, as well as the NAC and the MADS-box transcription factors could be conserved in both processes, as some introductory works seem to indicate (Pinyopich et al., 2003; Moreno-Risueno et al., 2010; reviewed in Benková and Bielach, 2010). Finally, downstream the auxin signaling cascades, the activation of cell cycle genes will take place in order to promote organ growth, as it is starting to be revealed in the case of LR (Rast and Simon, 2008). Thereby, the analysis of the expression of cell cycle genes during ovule primordia formation would be very revealing. Apart of the hormonal and molecular pathways controlling LR formation, the influence of the environmental factors on this process is of extreme importance for the plant. How environment influences ovule primordia formation would be for sure a very challenging topic of research.

With this work we wanted to point out the little specific information available about the factors that control ovule primordia initiation, due to the difficulties to identify mutants presenting defects only in this particular step of ovule formation. Here we propose different experimental approaches to overcome the severity of some mutant phenotypes as well as to investigate these processes from a new point of view. The contribution and conservation of chromatin remodeling changes to the regulation of ovule number is starting to be elucidated and opens an extremely interesting field of research. Moreover, the most recent progresses in the fields of ovule, flower and root development strongly suggest common hormonal and molecular signals in all these organ initiation processes; such as a crosstalk between auxin and CK and probably also BR and the factors that establish organ boundaries and those that promote new organ outgrowth.

### Conflict of interest statement

The authors declare that the research was conducted in the absence of any commercial or financial relationships that could be construed as a potential conflict of interest.
